# Gemfibrozil as a potential heme-independent sGC activator

**DOI:** 10.1186/1471-2210-11-S1-P68

**Published:** 2011-08-01

**Authors:** Michael Sobolevsky, Swati Chauhan, Iraida Sharina, Emil Martin

**Affiliations:** 1 Univeristy of Texas Houston Medical School, Department of Internal Medicine, Division of Cardiology, Houston, Texas, USA

## Background

Cyclic GMP producing soluble guanylyl cyclase (sGC) is highly activated by the binding of nitric oxide (NO) messenger to its heme. Besides NO, other NO-independent molecules were shown to activate sGC. These include a class of heme-replacers (e.g., protoporphyrin IX and structurally unrelated activators BAY 58-2667 and HMR1766) or sGC stimulators (e.g., BAY 41-2272 and YC-1 and aryl-acrylamide derivatives). The later class requires heme and sensitize sGC to low NO concentrations. Collectively these data suggest that various modes of allosteric sGC regulation are possible and some may have not been identified yet.

## Results

We screened a library of “off-patent” drugs and identified gemfibrozil (dimethylphenoxy-dimethylpentanoic acid), an anti-hyperlipidemic fibrate, as NO-independent sGC activator. Other tested fibrates (fenofibrate, clofibrate, bezafibrate, ciprofibrate) did not activate sGC. Structure-activity studies using molecules with phenoxy or dimethylpentanoic acid groups identified gemfibrozil moieties strictly necessary for sGC activation. In good agreement with the data on purified sGC, gemfibrozil dose-dependently relaxed preconstricted rat aortic rings. Consistent with direct sGC activation, endothelium denudation did not affect the vasorelaxing properties of gemfibrozil. Some of the sGC-activating gemfibrozil analogues, also showed a slightly improved vasorelaxing properties.

By evaluating the effect of gemfibrozil on the activity of truncated catalytically active sGC mutants we determined that gemfibrozil requires the heme-binding domain of sGC for its function. Enzymatic studies showed that, in contrast to heme-dependent stimulators BAY 41-2272 and YC-1, gemfibrozil does not affect the EC50 for NO, but diminishes the extent of sGC activation by NO. In addition, gemfibrozil-dependent activation is additively enhanced by allosteric regulator BAY 41-2272, suggesting a non-overlapping binding site. Gemfibrozil-dependent activation of sGC is stimulated by the depletion of heme, analogous to the heme-replacing BAY 58-2667 and HMR1766. However, in contrast to heme-independent stimulators the extent of activation is not affected by oxidation of sGC heme. Competition data suggest that gemfibrozil binding site may overlap with one of the heme-replacing stimulators.

## Conclusion

These data indicate that gemfibrozil represents a new class of sGC activators which may have a distinct mode of action. These data also indicate that besides anti-lipidemic properties gemfibrozil and some of its analogues may also affect vascular plasticity.

